# Romance and Reality in Married Life

**Published:** 1914-06

**Authors:** Charlotte Perkins Gilman


### Romance and Reality in Married Life.

BY CHARLOTTE PERKINS GILMAN.

(Reprinted from *Physical Culture* by permission.)

It has been said that "Hell is paved with good intentions." Some part of that conveniently illustrative region must be also rendered prickly and uncomfortable with shattered honeymoons.

There was a picture in *Punch,* long years ago, of a sad, bored man lounging upon a beach at some shore resort by the side of a sad, bored woman, idly twirling her parasol. "Don't you wish some friend would come along?" said she. And he answered: "Yes—or even an enemy."

These people did not *love* each other. Love is never sated, never tired.

The fleeting nature of passion is symbolized by giving wings to the little blind god, that creature of such visible limitations, under whose erratic guidance the world has so long wandered.

Cupid is not the god of love, not love in any noble sense. He is the god of desire, of sex attraction, a perfectly honorable and useful feeling, serving a great purpose in the universe—but not Love.

Cupid, shooting blindly, stirs bird and beast to the same swift passion as ourselves, and, fluttering on his way, is soon gone from them, as from us.

Do they object and complain? Not so far as we can see. They forget it and go on grazing—if they are grazing creatures. They are soon immersed in the cares of parentage if they are the species who unite in providing for the young.

Why, then, do we object and complain? What have we sought to counter-balance this fleeting nature of Cupid by chaining up the woman so that she cannot fly—without drastic penalties, and allowing the man to do his flying—under cover?

As we have become more and more human, higher love has come to us, a love which leaves poor Cupid hopelessly in the background, a love which includes passion, but transcends and outlasts it.

This love is not blind, and its wings are for long and steady soaring. It has no devious, zigzag butterfly flight, but holds one steady course. It grows with years, deepens, strengthens, sweetens, as time passes.

When the honeymoon is over, and two human beings, reared in quite different ways, having few common interests, and often lacking mutual understanding, find themselves married—but no longer "in love"—what are they to do?

It is a trying position, if they have only Cupid to hold them together, and no higher love.

Everything in the surroundings of the woman has been directed to intensify her femininity. Most things in the man's environment have tended to emphasize his masculinity. He has this advantage, however, among many—that of a larger development of humanness. For instance, he has his work, which is a human characteristic, not a male one. He has a larger area of brain surface in use, not being necessarily more "cultured"—frequently less so; but the world he inhabits is generally a larger one than hers.

Even if she is better educated, in our narrow sense, that means more knowledge of books and pictures and music perhaps, but he knows more—far more—of business, politics, the large movements of the world. Also she merely "knows" her things; he "does" his. He has associates, friends, comrades and fellow-workers. She has friends and acquaintances—with whom she exchanges calls.

When "love has flown" from the man, he has all the rest of life left—just as it was before he met his chosen lady. How about her? Reared from infancy to be as feminine as possible, having many fancies and few facts about "love," expecting of it a continuous flood of wonder and joy, she has entered upon a new life in which he is the center and circumference—the beginning and the end.

If he feels "she no longer loves me," he goes out into the world, takes up his work, forgets his disappointment—at least temporarily—in crowding occupation.

When she feels "he no longer loves me," she looks at the door he has shut behind him—the door that shuts her in—and goes back to her kitchen, or parlor, alone.

Housework occupies one's hand, but not always one's mind. A new home, with a young bride all alone in it, and Cupid temporarily gone, is a very lonesome place.

Yet sooner or later, to many and many a married pair, that melancholy feeling comes. The "first fine careless rapture" is quite gone; the thrill, the excitement, the *couleur de rose*—all gone.

Then what are we to do?

Now let us suppose the case to be a purely average one. Our young couple are not hopelessly mismated, so that every year of living together will but widen the breach between them, and make them more utterly miserable. They are not mismated—only misadjusted. Something wrong with the harness perhaps, or the management.

What is the cause of their distress—usually?

A very simple one—over-indulgence. Like a greedy child filling his cup half full of sugar and finding to his surprise that a drink could be too sweet; like any glutton who has eaten so much that he loathes all food; so does many a man spoil his own married happiness by trying to enjoy too much.

Any natural appetite indulged to excess results in satiety and then repugnance. One may be fond of quails, but the "quail a day" test has spoiled that taste for many a man.

Even more blamable than this is the unthinking cruelty with which a man who knows enforces his demands upon a timid, shinking bride who does not know. She loves him, but love speaks to her in different terms. She needs patience, teaching, wooing. What might have been a glad acceptance is made a passive submission or an active horror—by mere brutal haste. A lover should always woo.

All this difficulty could be avoided by proper teaching to both boys and girls, and by a little patience, courtesy, kindness, added to that blind youngster's arrows. But waiving both these avoidable causes, let us suppose that our two people, with all decent patience and restraint, still come to a stretch of plain pasture-land with no roses about it—still find that the glow of their attraction has cooled. Then what are they to do?

They need the help of two great powers, the greatest powers in life—Reason and Love. By the light of the one they may understand their difficulties. By the force of the other they may overcome them. Let them reason thus: (If they are still too much attracted, or repelled, to reason together, let them each work it out separately.)

"Here I am, married. There is no real cause for wishing it undone—much less for undoing it. But it is not what I hoped —I am unhappy. My wife (husband) no longer loves me. * * * In what way may I amend the position so as to be happier?"

This question is not hard to answer. What makes people happy who must live with some other person, is to love that person.

"But she (he) no longer loves me!" That is not the point. One can love even when the other does not. Besides that is not love at all—that "no longer" kind. It is merely Cupid, who has "flown," perhaps—why shouldn't he? When shall we learn that the quivering heights of joy do not constitute a level plain?—if they did they would cease to be heights.

You cannot look at the noblest picture continuously. You can not listen to the most exquisite music continuously. You can not hold the flavor of the most perfect peach continuously. Nobody expects to.

But, singularly enough, we do expect to hold at a steady level that which is in its very nature periodic, increasing to a climax and then ceasing. Of course our great trouble here lies in not recognizing that periodicity. Man has sought to transcend the laws of Nature, and make to himself a love-world in which the roses shall have no thorns and shall bloom all the time. He has succeeded in developing the thorniest kind of roses, most unreliable in their blooming, while the other creatures go on peacefully obeying—and enjoying—the laws of their nature.

Cupid, we agree, has temporarily departed.

What remains?

The man you are married to remains, or the woman you are married to; the partner in parentage, the chosen friend, the companion for life.

Any human being not actually repugnant may be loved, and that by the simplest of processes—by serving them.

If, for the time being, the husband or the wife no longer rouses in the heart of the other that sweet excitement of the first months of marriage, they are still human beings, still companions, and there is far more to loving than that one interchange.

The husband is in many cases less to be pitied than the wife, because if she is faithful to him and serves him properly in the housework, he does not suffer as keenly as she does in mere lack of affection.

Some wives, again, if they are well provided for in home and clothes and servants, do not seem to feel that heart hunger particularly.

But most of us, men or women, are alike in our longings to be loved.

This longing begins in childhood. It means at first the baby's need of the mother, and that need grows throughout life. But this love we long for has nothing to do with Master Cupid. It is the love that tries to make its object happy, tries to give comfort, benefit, service.

So when one feels this terrible conviction that the other no longer loves, the right way is to turn the question on oneself—"Do I love him (or her) as I should?" This is not the same thing as saying: "Do I want him—or her?" Wanting is not loving. Love is an outgoing feeling, not a mere vacancy. If you love anyone your object is to give happiness—not to demand it.

Hide your heart-hunger, if you have one; fast a little—it will not kill you; turn to and learn how to so surround the other with care, comfort, consideration, so that presently, to your pleased surprise, you find you have won over again the very thing you wanted.

This service should not be slavish. No man can really love a doormat, though he may find it useful; and a woman is even less impressed by a grovelling devotion. If you wish to win love you should show those qualities the other most admires—if you can. It is of no use to say: "But he used to love me when I never did a thing for him"—or "When I was much more foolish than I am now." That is not to the point. Then he was "in love." What we are discussing is the time when he isn't—and you wish to build a deeper, more lasting feeling.

If one sits hopelessly longing for something that is wholly gone, that is sheer misery. But if one feels there is gold in the ground to be dug out, or a crop to be raised if one ploughs, plants, and cultivates, then one may take heart of grace and fall to work.

Let the man say: "I will be just as strong and fine a man as I am capable of being. I will deserve her love by steady growth and improvement. I will win her love by intelligent tender care for her, by making her so comfortable and happy that she cannot do without me." Let the woman say the same thing, from her side.

It is no such desperate problem—to win the affection of human beings.

Study them. Try to understand them. Learn their real needs and try to meet them. And all the time live up to one's best ideals.

Beyond all this we should remember that love is not the whole of life—even for a woman. It is easier for a man to bear the lack of it because he has a wider life outside, but she also may strengthen herself by taking an honest interest in work, in study, in any line of human growth that is open to her.

If one gains the love one's heart desires, then all the other gain is added to it; and if not—it is better to be a strong, noble, useful person, even without love, than to be without love—and to have nothing else!

All life remains. All its interests, pleasures, activities, hopes, fears, dangers, excitements.

The greatest joys of life are human work and human love. As to work, the man has his, though often belittled by his arbitrarily limiting its purpose to filling his own pockets.

But what shall she do whose work is expected for the most part to consist in house-service? Why, if she can do that work well—or any part of it, let her make it a big, healthy world-business with a salary. Let her make a great name for herself, for her service, in lifting the standards of laundry work for her city, or of bread-making, or of sewing or cleaning, or what she prefers. If none of these are really her work then let her have the joy of striking out in new fields, learning the business that belongs to her, and doing it proudly.

If temporary conditions absolutely forbid such change, if she must keep on doing work she is unfit for, then she must place her reliance on the other great power—Love.

Real Love, this, not Cupid. The love which is in the human heart, in unsounded depths, and is to be brought out into consciousness and joyful growth by merely using it.

Love grows with service. Love grows with study, with interest, with the effort to understand, to help, to benefit.

A human being is an interesting study. Unless there is absolute repulsion, the mere process of living together, rightly fulfilled, should bring a growing happiness. It is not hard to please people, to win confidence, respect, affection. Most of us are pathetically grateful for understanding and sympathy.

If there are children as a common interest, the case is in some ways easier. If not, there is more time and strength to give to the beautiful work of winning a human heart—not merely a body.

Make believe that this person who "no longer loves you" is not required to, and do your turn about and love him—or her—as a dear friend. Then there knits and grows from day to day between the two whose lives are so close-neighbored, all the warm fabric of mutual experience, mutual gratitude for kindness and thought, mutual forbearance in small offences, mutual respect, patience, and understanding.

This kind of love does not "fly." It roots and grows like a tree. It has a solid foundation. It increases from year to year. It does not depend on youth, beauty, or fine clothes. It is sure and safe and lasting. And sometimes, under the shelter of this greater love, small Cupid flutters in and out, welcome enough, but not too hopelessly missed.

---

The Purdue Frederick Co. are sending out blotters with the suggestion that physicians specify an *"original package,"* and thus prevent the druggists from jeopardizing the lives of their patients by putting up an inferior preparation of his own make. A good suggestion this.

---

Walter B. Cannon, Chairman, Bureau for the Protection of Medical Research of the American Medical Association, declares that much of the hostile criticism against animal experimentation in medical research work grows out of the fact that the doctors reporting these experiments fail to state that an anesthetic was used. He suggests that in every instance in which anesthesia is a condition in the investigation, and especially in clinical articles which discuss new and unusual methods of diagnosis and treatment, that the doctor shall make clear the facts that the methods used were undertaken with the consent of the patient or his relatives, or, in the case of children, with the full consent of the parent.

This is a wise suggestion, and no intelligent physician will fail to appreciate and act upon the suggestion.

